# Veno-Arterial Extracorporeal Membrane Oxygenation as a Bridge to Heart Transplant—Change of Paradigm

**DOI:** 10.3390/jcm11237101

**Published:** 2022-11-30

**Authors:** Dubravka Šipuš, Kristina Krželj, Željko Đurić, Hrvoje Gašparović, Davor Miličić, Jadranka Šeparović Hanževački, Daniel Lovrić

**Affiliations:** 1 Department of Cardiovascular Diseases, University Hospital Center Zagreb, Kišpatićeva 12, 10000 Zagreb, Croatia; 2 Department of Cardiac Surgery, University Hospital Center Zagreb, Kišpatićeva 12, 10000 Zagreb, Croatia

**Keywords:** extracorporeal membrane oxygenation, heart transplantation, heart failure

## Abstract

Despite advances in medical therapy and mechanical circulatory support (MCS), heart transplant (HT) remains the gold standard therapy for end-stage heart failure. Patients in cardiogenic shock require prompt intervention to reverse hypoperfusion and end-organ damage. When medical therapy becomes insufficient, MCS should be considered. Historically, it has been reported that critically ill patients bridged with veno-arterial extracorporeal membrane oxygenation (VA-ECMO) directly to HT have worse outcomes. However, when the heart allocation system gives the highest priority to patients on VA-ECMO support, those patients have a higher incidence of HT and a lower incidence of death or removal from the transplant list. Moreover, patients with a short waiting time on VA-ECMO have a similar hazard of mortality to non-ECMO patients. According to the reported data, bridging with VA-ECMO directly to HT may be a solution in the selection of critically ill patients when the anticipated waiting list time is short. However, when a prolonged waiting time is expected, more durable MCS should be considered. Regardless of the favorable results of the direct bridging to HT with ECMO in selected patients, the superiority of this strategy compared to the bridge-to-bridge strategy (ECMO to durable MCS) has not been established and further studies are mandatory in order to clarify this issue.

## 1. Introduction

Heart failure is one of the leading causes of morbidity and mortality with a prevalence of 1–2% among the adult population in developed countries [1]. The incidence and prevalence of heart failure are constantly increasing due to the prolonged human lifespan. Despite advances in medical therapy and mechanical circulatory support (MCS), heart transplant (HT) remains the gold standard therapy for end-stage heart failure [2]. A persistent disproportion of available donor hearts and recipients and long waitlist times have resulted in a significant increase in the number of patients bridged to HT with MCS. Durable continuous flow ventricular assist devices (VAD) are used as a bridge to HT with the aim of improving the survival rates of patients with end-stage heart failure, and patients with implanted durable VAD do have similar survival rates as those with primary HT [3,4,5]. However, favorable clinical outcomes are achieved when durable VADs are implanted in stable patients with isolated left ventricular failure (left ventricular assist device —LVAD) [6,7], whereas VAD implantation is not recommended in patients with cardiogenic shock. 

Unlike the use of durable LVAD as a bridge to HT, the use of ECMO as a direct bridge to HT, although increasing [8,9], is controversial. There is some evidence that patients bridged with ECMO have worse outcomes after HT [9,10,11,12], which may be explained by reduced time for end-organ damage recovery and the inability to undergo comprehensive pre-transplant assessment [10]. This review will present new findings regarding the increasing use of ECMO as the bridge to HT strategy in patients with end-stage heart failure in different donor organ allocation systems in the USA and Europe.

## 2. Cardiogenic Shock and VA-ECMO

Cardiogenic shock is a physiologic state of inadequate tissue perfusion resulting from inadequate cardiac output due to cardiac dysfunction. Cardiogenic shock manifests as hypotension (systolic blood pressure < 90 mmHg) and unresponsiveness to volume resuscitation with a cardiac index below 2.2 L min^−1^ m^−2^ and pulmonary capillary wedge pressure ≥15 mmHg. Tissue hypoperfusion results in metabolic acidosis and elevated serum lactate and creatinine level [2,11,12,13]. Despite all advances in pharmacological therapy and mechanical circulatory support, in-hospital mortality of cardiogenic shock remains high (27–51%) [12]. 

The Society for Cardiovascular Angiography and Interventions (SCAI) classified cardiogenic shock into five stages of increasing severity (from A to E) (Table 1) [14]. This classification represents a simple and clinically applicable system from pre-hospital providers to intensive care units [14]. Consistent use of cardiogenic shock classification allows the appropriate comparison of outcomes between patients in different cardiogenic shock categories and contributes to clinical decision-making. According to Extracorporeal Life Support Organization recommendations, ECMO should be considered when the mortality risk with conventional medical therapy is >50% (stage D) [15]. VA-ECMO may be used as a bridge to recovery or to a definitive treatment such as durable VAD or HT. If there is insufficient data on the patient or the disease, ECMO could be a reasonable choice to allow additional time for adequate diagnostics, stabilization of the patient, and finally, to arrive at the most beneficial decision on definitive treatment [13,16].

## 3. ECMO as a Bridge to HT Strategy in Different Allocation Systems—Analysis of Outcomes

The majority of available data on bridging strategies to HT comes from retrospective analyses of The United Network for Organ Sharing (UNOS) database. Several analyses stemming from the UNOS database have shown the poor survival of patients who were bridged to HT with ECMO [10,17,18]. A multivariable analysis by Moonsamy et al. showed that ECMO support before HT was an independent risk factor for mortality, confirmed by a propensity match analysis [10]. Despite this fact, during the period from the year 2006 to 2019 the percentage of transplant recipients bridged with ECMO increased from 0.6% to 4.9% [5]. Later transplant years had even shown the protective effects of ECMO, suggesting that there is a learning curve in the temporary MCS [10]. Furthermore, in October 2018 the UNOS implemented a new six-tier heart allocation system with the goal of broader and more equitable organ sharing in the United States. The consequence of that change was a significant increase in the number of patients bridged to HT with ECMO or other types of temporary MCS [19]. The major differences between the old and new allocation system are shown in Table 2.

Gonzalez et al. compared the outcomes of patients with ECMO support listed for HT in the old (2015–2018) and new allocation system (2018–2019). Patients listed in the new allocation system had a significantly higher cumulative incidence of HT, a lower incidence of death or removal from the transplant list and a shorter median waitlist time on ECMO support (three days vs. seven days, *p* < 0.006). Furthermore, the six-month post-transplantation survival was 74.6% and 90.6% for old and new-era patients, respectively (*p* = 0.002) [19]. A similar study was performed by Hess et al. who demonstrated a higher likelihood of HT in patients supported with ECMO in the new allocation system and a marked reduction in median waitlist time from 47 to four days. The authors established comparable one-year survival rates in the new and old system (new 79.8% vs. old 90.3%; *p* = 0.3917), with equivalent rates of stroke and one-year acute rejection, although postoperative renal failure was significantly higher in the new policy group [22]. Several other studies reported significant improvement in post-transplant survival, a lower hazard of post-transplant mortality, a lower rate of waitlist mortality, and an up to 50% reduced waitlist time after implementation of the new system (from 11 or 10 days vs. five days) [23,24], as well.

In Spain, listing for high-urgent HT is allowed for critically ill patients who cannot be weaned from temporary MCS. Due to economic restrictions on access to durable LVAD and the quick availability of donors, the use of temporary MSC as a direct bridge to transplant is the most common mode of bridging and even allows the exploration of different short-term support strategies. Barge-Caballero et al. conducted a retrospective analysis of adult patients listed for high-urgent HT under temporary devices from 2010 to 2015 in 16 Spanish institutions. The authors compared outcomes of patients supported by VA-ECMO, temporary LVAD (T-LVAD) and temporary biventricular VAD (T-BiVAD). They showed that T-LVAD support was independently associated with a lower risk of death over the first year after listing for HT than the other two support methods (postoperative mortality VA-ECMO 33.3%, T-LVAD 11.9%, T-BIVAD 26.2%, *p* = 0.008). The high mortality of HT candidates supported with ECMO found in this study may be explained by the fact that these patients were in more critical condition, and with an observed higher incidence of adverse clinical events associated with temporary MCS. However, these patients experienced significantly shorter time from device insertion to the high-urgent listing (3 ± 5 days vs. 11 ± 14 for T-LVAD and 7 ± 9 days for T-BiVAD, *p* < 0.001), most likely because ECMO support was the least durable of all three [25]. 

In France, patients on VA-ECMO have had the highest priority since 2004. According to the analysis of the French national registry CRISTAL, Jasseron et al. [7] reported a lower one-year overall survival for patients bridged with VA-ECMO (52.2%, 95% CI, 40.5–62.6%, controls 75.5% 95% CI, 72.4–78.4%; *p* < 0.01). It is important to note that in this study, the patients in the ECMO group were significantly more likely to be on intravenous inotropes, mechanical ventilation, dialysis and had a higher serum bilirubin level. Moreover, authors found a lower risk of mortality in VA-ECMO patients after HT (hazard ratio, 0.44; 95% CI 0.2–0.9), suggesting the survival benefit of HT in patients on VA-ECMO who were eligible for listing [7]. A large observational single-center retrospective study by Coutance et al. established, however, similar post-transplant survival rates of patients listed on VA-ECMO support and non-VA-ECMO patients (85.5% and 80.7% respectively, *p* = 0.12) [26]. Several smaller studies have reported good outcomes in patients bridged to transplantation with VA-ECMO support as well [27,28]. 

In the United Kingdom, the donor allocation system changed in 2016, giving the highest urgency to patients on temporary MCS. After the change, waiting time for patients on temporary MCS was significantly shorter with a significant decrease in deaths on the waiting list (5% to 2%). The one-year post-transplant survival of patients on temporary MCS was comparable to the survival of patients from other categories. It is worth noting that the number of non-urgent transplants was unaffected [29]. However, bridging with VA-ECMO in the United Kingdom is rare, and Rushton et al. reported only eight patients bridged with VA-ECMO directly to HT after the change of allocation system [30].

Although there are certain differences in the allocation systems of the four western countries mentioned above (Table 3.), the most important common criteria to note are the priority status of ECMO patients for HT [21,31,32,33] and a relatively short time allowed to remain in the highest urgency group, ranging from seven (USA, Spain) [21,31] to a maximum of 16 days (France) [32] (Table 3). The application of these specific criteria is most likely the cause of the improved outcomes observed among candidates for HT supported with ECMO in these four countries.

## 4. Prediction of Outcomes in Patients with VA-ECMO Support as Bridge to Heart Transplant—A Comparison of Scoring Systems

Many authors have analyzed potential risk factors for poor outcomes and evaluated pre-existing scorings systems to determine their potential utility in predicting eventual suitable candidates for bridging to HT with VA-ECMO.

Cho et al. [28] compared two comprehensive scoring systems for organ failure with the duration of MCS in predicting survival after HT. The authors compared the Sequential Organ-Failure Assessment (SOFA) score [34] and the Model for End-stage Liver Disease (MELD) score modified by the UNOS, the so-called MELD UNOS score.

The MELD score was created to predict liver disease severity and includes serum creatinine level, serum total bilirubin and international normalized ratio (INR). In 2002 the UNOS was incorporated into the MELD score to make the MELD UNOS score so as to prioritize patients on the waitlist for liver transplantation. Unlike the MELD score, the SOFA score involves the status of six major organs (the lungs, brain, heart, liver, kidneys, and bone marrow) but has several confounders in this setting. Pulmonary function in SOFA score is assessed using blood sampled from the radial artery, but arterial blood gas analysis in patients with severe heart failure on VA-ECMO is not a good reflection of pulmonary function. Furthermore, platelets number used to reflect bone marrow function cannot be used in those patients whose bone marrow is affected by frequent transfusion or in those with MCS-induced thrombocytopenia [28]. The authors established that MELD UNOS score was an independently better predictor of death after HT compared with the duration of MCS and the SOFA score. The cut-off value for the MELD UNOS score of 24 had the highest sensitivity (86% (95% CI) (42.1–99.6)) and specificity (83% (95% CI) (58.6–96.4)) in comparison with cut-off values of MCS duration (five days; (95% CI), sensitivity 86%, (42.1–99.6), specificity 56%, (30.8–78.5)) and SOFA score cut-off value of 13, ((95% CI), sensitivity 86% (42.1–99.6), specificity 83% (41.0–86.7)). In patients with a MELD UNOS score > 24, the expected one-year survival was 33% compared with 91% in patients with a MELD UNOS score ≤ 24 [28].

The findings from the study by Cho et al. [28] correlate with the observation by Fukuhara et al. who reported that the MELD score excluding the INR (MELD-XI score) is the sole contributor to both 90-day and three-year mortality. The authors observed that ECMO patients with MELD-XI score >17 had poorer post-transplant survival than those with a MELD-XI score <13 (90-day, 54.4% vs. 85.0% (*p* < 0.001) and three-year, 49.5% vs. 73.5% (*p* < 0.001]) [18].

The in-hospital mortality assessment tool for critically ill patients—the Acute Physiology, Age, and Chronic HEalth evaluation IV (APACHE IV) score [35]—is another score that could be used to predict survival in patients bridged with ECMO to HT. Lechiancole et al. established that an APACHE IV value of 47 (specificity of 84.6% and sensitivity of 100%) delineates between a group with low (Group A < 47), and high (Group B ≥ 47) probability of 30-day mortality. Reported 30-day mortality in group B was 60%, whereas one- and five-year survival probability in Group B was 26.6%. In Group A no early mortality was observed and the estimated survival was 89.7% at one year and 81.5% at five years, respectively [36].

## 5. Discussion

Patients in severe cardiogenic shock require immediate intervention to reverse hypoperfusion and end-organ damage. VA-ECMO is an accessible MCS, easily inserted percutaneously by the Seldinger technique and provides full cardiopulmonary support. Therefore, the use of VA-ECMO in cardiogenic shock constantly increases [13,19]. 

However, critically ill patients bridged with VA-ECMO directly to HT have shown worse outcomes. Most of these findings were based on reports from the USA before implementing the new six-tier heart allocation system in October 2018. Before the allocation system change, critically ill patients on VA-ECMO support had to compete for organs with stable LVAD patients, patients supported with an intra-aortic balloon pump (IABP), or those on high doses of single or dual inotrope therapy [19,37]. Under the new allocation policy, stable and dischargeable patients with LVAD became less urgent in comparison with those on ECMO and IABP who became prioritized. Unlike the USA, in some European countries (Spain, France, United Kingdom), higher priority to ECMO patients was present for a longer period.

The authors from centers in the USA that compared outcomes of patients bridged to HT with VA-ECMO in new and old allocation systems reported higher cumulative incidence of HT and lower incidence of death or removal from the transplant list after allocation system change [19,22,23,24]. Furthermore, post-transplantation survival rates were similar or even higher for patients listed after the allocation change, probably due to a significant reduction in waitlist time. Some authors have even reported that, after the change of allocation policy, patients bridged with VA-ECMO directly to HT had a similar risk of mortality when compared with the non-ECMO cohort [22,23]. Reports from European countries that assigned the highest priority to patients on VA-ECMO listed for HT brought similar results as those published in the USA—patients bridged to HT with VA-ECMO have similar survival rates as non-VA-ECMO patients [25,26,27,28].

Giving the highest priority on the HT waitlist to the most critically ill patients, as ECMO patients are, is the most important common criterion of the allocation systems in the USA, Spain, France and the UK. Implementation of such allocation systems has brought encouraging results among the patients bridged to HT with ECMO because the incidence of HT became significantly higher, and waitlist time, as well as the time for ECMO-related complication development, became significantly shorter. It is worth noting that the provision of care for such patients in experienced tertiary centers results in fewer ECMO-related complications, additionally contributing to better survival rates after HT [38,39,40].

However, there are many challenges regarding the strategy of using ECMO as a bridge to HT. Two important questions are who could be considered a suitable candidate for the ECMO bridge to HT and how to accurately predict the survival of the candidate elected for this strategy. There are several factors that have been found to influence the survival rate in patients on ECMO support, such as mechanical ventilation [10], creatinine level [5,40], bilirubin level [29], the need for dialysis [10], pulmonary and brain function [36], bone marrow function [36], etc. However, the reported results are inconsistent, emphasizing the need for a more comprehensive and accurate scoring system. Encouraging results were obtained for MELD UNOS [29], MELD-XI [18], and APACHE IV [36]. These scores have high specificity and sensitivity to predict mortality in patients bridged to HT with ECMO and may be helpful in decision-making. However, consistent application of these scores among patients bridged to HT with ECMO is required in order to obtain invaluable databases for the delineation of categories with assumed better or worse outcomes.

The third open question in clinical practice is whether it is preferable to use ECMO as a direct bridge to HT or as a bridge to a durable LVAD. DeFilippis et al. reported that ECMO use as a bridging strategy to LVAD in the United States increased significantly over time from 0.0% to 5.1% (2006 to 2017). This study established no difference in mortality on pump support compared with post-transplant mortality among those bridged with ECMO [5]. It is important to emphasize that not all critically ill patients on ECMO support are suitable candidates for LVAD implantation, as the best clinical outcomes were observed when durable LVAD was implanted in stable patients with isolated left ventricular failure [6,7]. However, if the patient has no absolute contraindication for HT, the evidence-based recommendations on whether to introduce the patient on ECMO support to the urgent HT waitlist or to switch the management towards LVAD implantation do not exist. This decision requires comprehensive discussion and consensus among heart team members in a specialized tertiary center.

## 6. Conclusions

Bridging with VA-ECMO directly to HT can be considered in selected critically ill patients when the anticipated waiting list time is short. Best survival rates are achieved with modifications of the donor heart allocation system in a way that the most critically ill candidates have priority, such as candidates with VA-ECMO support. There are many scores that may help evaluate appropriate candidates for the VA-ECMO bridge to HT, such as MELD-UNOS, MELD-XI and APACHE IV scores. It is important to note that survival rates among VA-ECMO patients, including those bridged to HT, are significantly higher in experienced centers. Though there are encouraging results of direct bridging to HT for a selected group of patients on ECMO, we cannot conclude that this strategy is superior to a bridge-to-bridge strategy (ECMO to durable VAD) and further studies are mandatory for the elucidation of this issue.

## Figures and Tables

**Table 1:** The Society for Cardiovascular Angiography and Interventions (SCAI) classification of cardiogenic shock into five stages of increasing severity (A–E) [14].

Stage	Description	Physical Exam/Bedside Findings	Biochemical Markers	Hemodynamic
**A** **At risk**	A patient who is not currently experiencing signs or symptoms of CS but is at risk for its development. These patients may include those with large acute myocardial infarction or prior infarction as well as those with acute and/or acute-to-chronic heart failure symptoms.	Normal JVP Lung sounds clear Warm and well perfused →Strong distal pulses →Normal mentation	Normal labs →Normal renal function →Normal lactic acid	Normotensive (SBP ≥ 100 mmHg or normal for patient.) If hemodynamic done →Cardiac index ≥ 2.5 L/min/m^2^ →CVP < 10 mmHg
**B** **Beginning CS**	A patient who has clinical evidence of relative hypotension or tachycardia without hypoperfusion.	Elevated JVP Rales in lung fields Warm and well perfused →Strong distal pulses →Normal mentation	Normal lactate Minimal renal function impairment Elevated BNP	SBP < 90 or MAP < 60 or > 30 mmHg drop from baseline Pulse ≥ 100 bpm If hemodynamic done →Cardiac index ≥ 2.2
**C** **Classic CS**	A patient that manifests with hypoperfusion that requires intervention (inotrope, pressor or mechanical support, including ECMO) beyond volume resuscitation to restore perfusion. These patients typically present with relative hypotension.	May include any of: Looks unwell Panicked, Ashen, mottled, dusky Volume overload Extensive rales Killip class 3 or 4 BiPap or mechanical ventilation Cold, clammy Acute alteration in mental status Urine output <30 mL/h	May include any of: Lactate ≥ 2 Creatinine doubling or >50% drop in GFR Elevated BNP	May include any of: SBP < 90 or MAP < 60 or >30 mmHg drop from baseline AND drugs/device used to maintain blood pressure above these targets Hemodynamic →Cardiac index <2.2 L/min/m^2^ →PCWP > 15 mmHg →RAP/PCWP ≥ 0.8 →Cardiac power output ≤ 0.6 W
**D** **Deteriorating/doom**	A patient that is similar to category C but is getting worse. They show a failure to respond to initial interventions.	Any of stage C	Any of state C AND: deteriorating	Any of stage C AND: Requiring multiple pressors or addition of mechanical circulatory support devices to maintain perfusion
**E** **Extremis**	A patient that is experiencing cardiac arrest with ongoing CPR and/or ECMO, being supported by multiple interventions.	Near pulselessness Cardiac collapse Mechanical ventilation Defibrillator used	‘Trying to die’ CPR pH ≤ 7.2 Lactate ≥ 5 mmol/L	No SBP without resuscitation PEA or refractory VT/VF Hypotension despite maximal support

Abbreviations: CS—cardiogenic shock; JVP—jugular venous pressure, SBP—systolic blood pressure, CVP—central venous pressure, BNP—brain natriuretic peptide, MAP—mean arterial pressure, GFR—glomerular filtration rate, PCWP—pulmonary capillary wedge pressure, RAP—right atrial pressure, CPR—cardiopulmonary resuscitation, PEA—pulseless electrical activity, VT/VF—ventricular tachycardia/ventricular fibrillation.

**Table 2:** The differences between the old and new allocation system in the USA.

USA Allocation Policy 1999–2018 [20]		USA Allocation Policy 2018—Present [21]	
**Status 1A**	MCS (VAD or total artificial heart or IABP or ECMO) for acute hemodynamic decompensation LVAD with complications (infection, thromboembolism, ventricular arrhythmias, mechanical failure, other related complications) Continuous mechanical ventilation Continuous single or multiple inotropes requiring hemodynamic monitoring Dischargeable LVADs for 30 days	**Status 1**	ECMO (<7 days) Non-dischargeable surgically implanted VAD MCS with life-threatening ventricular arrhythmia
		**Status 2**	IABP (<14 days) Sustained VT/VF Non-dischargeable, surgically implanted, non-endovascular LVAD (<14 days) MCS with device malfunction/mechanical failure Total artificial heart Dischargeable BiVAD or right ventricular assist device Acute endovascular percutaneous circulatory support (<14 days)
		**Status 3**	Dischargeable LVAD (< 30 days) Multiple inotropes or single high-dose inotropes requiring continuous hemodynamic monitoring MCS with complications (device infection, hemolysis, pump thrombosis, right heart failure, mucosal bleeding, aortic insufficiency) ECMO after seven days or any other temporary MCS after 14 days
**Status 1B**	All LVADs Continuous inotrope infusion	**Status 4**	Stable LVAD candidates not using 30-day discretionary period Inotropes without hemodynamic monitoring Congenital heart disease Ischemic heart disease with intractable angina Hypertrophic cardiomyopathy Restrictive cardiomyopathy Amyloidosis Re-transplant
		**Status 5**	Combined organ transplants
**Status 2**	All other listed candidates	**Status 6**	All other active candidates

**Table 3:** The comparison of highest priority groups between the contemporary allocation systems in the USA, Spain, France and the United Kingdom.

USA ^a^ [21]	Spain ^b^ [31]	France ^c^* [32]	United Kingdom ^d^ [33]
HT candidates on ECMO (maximum time of stay for these patients in the highest urgency grade is <7 days with possible prolongation for additional 7 days.) HT candidates with non-dischargeable surgically implanted VAD HT candidates with MCS device and with life-threatening ventricular arrhythmia	HT candidates dependent on temporary MCS and cannot be weaned off the device HT candidates with durable VADs and with complications (infection, pump failure, or thrombosis) The patients with ECMO or any temporary MCS offering partial support must be on the MCS a minimum of 48 h before entering the highest urgency status list and only provided they do not present criteria of multi-organ failure. The maximum time of stay for these patients in the highest urgency grade is <7 days.	HT candidate with the highest national score. The allocation system is based on a national score, going from 0 to 1151 and ranking all candidates. The candidate risk score (CRS) is the cornerstone of the allocation score. CRS includes VA ECMO use, plasma concentrations of natriuretic peptides, glomerular filtration rate (GFR) and total serum bilirubin level; CRS is generated by summing the products of each predictor multiplied by its coefficient Exceptions: Nine hundred points are allocated immediately or over a three-month period to patients on durable VAD with device-related complications and to patients on uncomplicated BiVAD and total artificial heart, as well as to those with sustained ventricular arrhythmia and to those with contraindications to durable VAD Time allowed for ECMO patients bridged to HT to stay in the highest priority group <12–16 days	HT candidate on temporary VAD or VA-ECMO support. HT candidate (a) on intra-aortic balloon pump (IABP) support (b) at imminent risk of death or irreversible complications. Meets criteria for urgent listing but is not suitable for long-term VAD

^a^ highest priority category—‘Status 1’; ^b^ highest priority category—‘Urgency status 0’; ^c^ highest priority category—highest national score; ^d^ highest priority category—‘Super-Urgent Heart Allocation Scheme’; * In the period 2004–2016 highest urgency status in France was reserved for HT candidates on ECMO support or those on intravenous inotrope infusion [32].
